# Effects of Partial Replacement of Sugar with Fig Syrup on the Survival of *Bacillus coagulans* and the Physicochemical Properties of Probiotic Ice Cream

**DOI:** 10.17113/ftb.61.03.23.8152

**Published:** 2023-09

**Authors:** Reza Janipour, Seyed Shahram Shekarforoush, Samira Ghorbani, Hamid Reza Gheisari

**Affiliations:** 1 Department of Food Hygiene and Public Health, School of Veterinary Medicine, Shiraz University, Eram Street, Shiraz, Iran; 2 Department of Food Hygiene and Public Health, School of Veterinary Medicine, Ardakan University, Ayatollah Khatami Street, Yazd, Iran

**Keywords:** dairy ice cream, *Bacillus coagulans*, fig syrup, sugar replacement

## Abstract

**Research background:**

Various sectors of the food industry demand the enrichment of food with functional compounds. Probiotic products with valuable nutritional and therapeutic properties have attracted great attention in the fields of industry, nutrition and medicine. The aim of the present study is to investigate the sensory and physicochemical properties of probiotic ice cream containing fig syrup and to evaluate the survival of *Bacillus coagulans* after 90 days of storage at -18 °C.

**Experimental approach:**

In this study, four experimental groups of ice cream were produced as follows: plain dairy ice cream (without additives), ice cream containing 10^9^ CFU/g *B. coagulans*, ice cream containing 25 % fig syrup as sugar substitute and ice cream containing 25 % fig syrup as sugar substitute and 10^9^ CFU/g *B. coagulans*. They were stored at -18 °C for 3 months. Texture, pH, acidity and viscosity were analysed and microbial counts were determined after 1, 30, 60 and 90 days of storage. The organoleptic evaluation was carried out on days 1 and 90.

**Results and conclusions:**

The results showed that during the initial freezing process and the transformation of the mixture into ice cream, the number of *B. coagulans* decreased from 10^9^ to 10^7^ CFU/g, without significant changes observed over the 90-day period. No significant changes were found in the sensory and textural properties of the samples either. Replacement of 25 % sugar with fig syrup reduced the pH, increased the acidity of the ice cream and improved their viscosity. In conclusion, the production of functional ice cream using fig syrup and *B. coagulans* is recommended for their health benefits.

**Novelty and scientific contribution:**

The results of this study can be used to prepare functional and healthy foods. Our results suggest that fig syrup has the potential to be used as a natural sweetener or sugar substitute in various products.

## INTRODUCTION

Nowadays, the introduction and promotion of functional foods have attracted more attention. Functional foods are considered to have health-promoting effects that go beyond those of a staple diet. These foods have demonstrated physiological benefits when consumed as part of the normal diet (*1*, *2*). Probiotics and prebiotics are functional ingredients used to enhance the health benefits of foods (*3*). Granato *et al*. (*4*) define probiotics as live microorganisms that are beneficial to the host when consumed in sufficient quantities. They have the potential to improve the immune system, modulate the intestinal microflora and inhibit the growth of pathogenic bacteria (*4*-*6*). Probiotic-enriched products should contain at least 10^6^ CFU/g of viable organisms to be considered healthy foods (*7*).

*Bacillus coagulans* is a spore-forming bacterium and belongs to a new family of probiotics. It has been compared to other probiotic bacteria such as *Lactobacillus* and *Bifidobacteria*. *B. coagulans* grows as Gram-positive rods, motile, single or rarely in short chains of variable length. Their optimal pH and temperature for growth are 5.5–6.5 and 30–50 °C, respectively. Metabolically, they are facultative anaerobic microorganisms that produce acid by fermenting maltose, mannitol, raffinose, sucrose and trehalose, without producing gas. The potential applications of *Bacillus* probiotics are not limited to dietary supplementation; they can also be used as a clinical therapy for gastrointestinal and urinary tract infections. Their therapeutic effect is mainly based on their ability to produce bacteriocins such as coagulin, which have a broad spectrum of activity against enteric pathogens (*8*).

Dairy products are the most common probiotic carriers (*4*). In this regard, ice cream is a suitable choice for the formulation of probiotic-enriched foods because it has a near-neutral pH that does not affect probiotics (*9*, *10*). Ice cream is rich in carbohydrates, milk proteins, essential amino acids, vitamins and minerals, and its ingredients are well absorbed by the body (*11*). However, consumer acceptance and survival of probiotics during storage should be considered when making a probiotic or symbiotic product. Two important criteria for the efficacy and success of probiotic and prebiotic products are consumer acceptance and the survival of probiotic microorganisms during production and storage (*12*, *13*).

Sucrose is an ingredient in ice cream that makes it tastier and more popular. Excessive sugar consumption increases the incidence of many diseases, such as dental problems, obesity, diabetes, high cholesterol and cardiovascular diseases. Therefore, consumers prefer alternative sweeteners instead of sucrose (*14*).

The fig is a sweet and nutritious fruit with numerous therapeutic properties. Figs are among the most valuable and energy-rich fruits consumed in various forms such as fresh, dried, canned, jam, syrup, concentrate, fruit jelly or chocolate and nut cookies (*15*). This fruit contains a variety of beneficial substances, including essential vitamins, antioxidants and antimicrobial and anticancer compounds. It helps lower blood sugar and fat levels (*16*). Therefore, figs can add special value to foods and be used in special diets such as low-fat, low-sodium, high-fibre, diabetic and mass-loss diets. Fig syrup has the potential to be used as a natural sweetener or sugar substitute in baked and cooked products. Compared to most fruits and vegetables, it is an excellent source of dietary fibre, which aids digestion (*15*). Therefore, the aim of the present study is to investigate the characteristic changes in ice cream after the addition of *B. coagulans* and partial replacement of sugar with fig syrup.

## MATERIALS AND METHODS

Before we started this project, we conducted a pilot study. In the production of ice cream, 25, 37.5 and 50 % of sugar was replaced by fig syrup. The ice cream with 37.5 and 50 % replacements did not have acceptable organoleptic and textural properties. As the 25 % replacement was acceptable, this amount of fig syrup was used instead of sugar.

In this study, four experimental groups of ice cream were produced as follows: plain dairy milk ice cream (control), ice cream with 10^9^ CFU/g *B. coagulans* as probiotic bacteria, ice cream with 25 % fig syrup replacement, and ice cream with 25 % fig syrup replacement and 10^9^ CFU/g *B. coagulans*. All productions were carried out in triplicate.

### Balancing the sweetness of the sugar with the fig syrup

To prepare fig syrup, dried white figs (*Ficus carica*) were obtained from the local market and after washing, they were mixed with tap water at a ratio of 1:3 and allowed to soak overnight. Then they were homogenised with a blender (model HR2291/01; Philips, Amsterdam, Netherands) and filtered with cheesecloth. Using a refractometer (model Dr-101; Cosecta S.A., Barcelona, Spain), the Brix value of the fig syrup was determined to 16° Brix at room temperature. According to the results of the HPLC analysis (1100 series; Agilent Technologies, Waldbronn, Germany), 100 mL fig syrup with Brix 16° contained 6.09 g fructose, 5.10 g glucose and 0.44 g sucrose. According to their sweetness coefficients (1.4 for fructose, 0.75 for glucose and 1 for sucrose), the sucrose equivalent of fig syrup (100 mL) was 12.8. To make 1 kg of ice cream with 25% sucrose replacement by fig syrup, 351.5 mL of fig syrup are needed to achieve the same sweetness as sucrose.

### Ingredients and probiotic strain

Khoshmaze Co. (Shiraz, Iran) supplied us with all the ingredients needed to make ice cream, including skimmed milk powder (34 % protein, 1 % fat; Pegah Infant Formula Co., Shahrekord, Iran), hydrogenated vegetable oil (Narges, Shiraz, Iran), commercial sugar (Bally, Isfahan, Iran), fig syrup as sweetener, vanilla (Polar Bear, Shanghai, China), carboxymethyl cellulose (E 466; Sunrose, Tokyo, Japan), panisol (E 471; Danisco, Copenhagen, Denmark), cellulose gum (E 410), guar gum (E412), carrageenan (E 407), tocopherol-rich extract (E 306) and ascorbyl palmitate (E304; Ramak Co, Shiraz, Iran). The probiotic *B. coagulans* strain in lyophilised form was kindly provided by Pardis Roshd Mehregan, Iran. Further details on the composition of the different experimental groups are given in Table 1.

**Table 1:** The composition of ice cream formulations

Ingredient	Treatment group			
Control	Fig *w*(ingredient)/%	Bc	Fig+Bc
Water	63.8	33.2	63.8	33.2
Skimmed milk powder	8.5	8.5	8.5	8.5
Carboxymethyl cellulose	0.2	0.2	0.2	0.2
Stabiliser - emulsifier	0.4	0.4	0.4	0.4
Vanilla	0.1	0.1	0.1	0.1
Hydrogenated vegetable oil	9	9	9	9
Sugar	18	13.5	18	13.5
Fig syrup	0.0	35.1	0.0	35.1
*Bacillus coagulans*	-	-	+	+

Control=plain dairy ice cream, Fig=ice cream with 25 % fig syrup instead of sugar, Bc=ice cream with *N*(*Bacillus coagulans*)=10^9^ CFU/g as probiotic bacteria, Fig+Bc=ice cream with 25 % fig syrup instead of sugar and *N*(*B. coagulans*)=10^9^ CFU/g

### Probiotic culture activation

Lyophilised probiotic *B. coagulans* was obtained from the Pardis Roshd Mehregan Company, Shiraz, Iran. The bacteria were activated by inoculation of the nutrient yeast extract salt medium (NYSM) broth culture at 37 °C for 24 h. The probiotic cells were centrifuged at 6000×*g* (Sorvall™ ST 8; Thermo Fisher Scientific Inc., Waltham, MA, USA) and then washed in sterile saline using the same centrifugation procedure. The probiotic bacteria were inoculated into the ice cream. The amount of the cells was adjusted to 10^9^ CFU/mL. The ice cream mixture was frozen at −4 to −5 °C and stored at −20 °C to harden (*2*).

### Ice cream production

The experimental mixtures were prepared in 3 kg batches. According to the recipes provided by the ice cream company (Khoshmaze Co.), the water was measured with a scaled cylinder, then the milk powder was added and heated to 40–45 °C. The other ingredients were then added to the reconstituted milk. After pasteurisation (85 °C, 15 min), the mixtures were stirred at 45 °C for 5 min using a simple mixer (model 6790; Tefal, Rumilly, France), cooled to room temperature and ripened overnight at 4 °C. They were then fortified with vanilla and a probiotic strain before freezing. The mixtures were whipped in an ice cream machine (model BQL-12Y; Shanghai Lisong, Shanghai, PR China) for 20 min at 52 rpm and frozen. The final products at a temperature of -5 °C were packed in plastic cups, hardened at -30 °C for 2 h and stored at -18 °C (*17*).

### Physicochemical analyses

The overrun (%) of the samples was measured using the following formula (*11*):


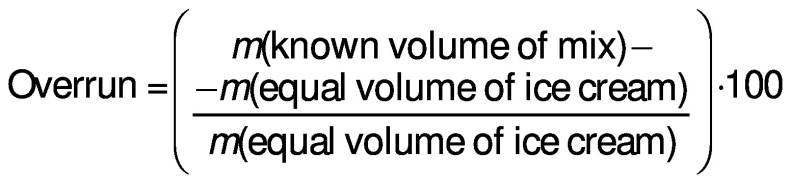
 /1/

After melting, pH was determined at room temperature using a digital pH meter (model 350; Jenway, Dunmow, UK).

For the determination of total titratable acidity, the sample was titrated with 0.1 M sodium hydroxide (NaOH) and a phenolphthalein indicator. The results were expressed as a percentage of lactic acid (*18*).

Viscosity was measured with a digital viscometer (model RVT; Brookfield Engineering Laboratories, Stoughton, MA, USA) at 25 °C.

The hardness, cohesiveness and adhesiveness of the samples were determined using a CT3 4500 texture analyser (Brookfield Engineering Laboratories) equipped with a stainless steel cylindrical probe (6.0 mm diameter, 35 mm height). Each sample was compressed twice to 50 % of its original height at a test speed of 2 mm/s.

### Probiotic count

The *B. coagulans* number of probiotic samples was counted on days 1, 30, 60 and 90 of storage. Serial dilutions of the samples were plated on nutrient yeast extract salt agar (Merck, Darmstadt, Germany) plates, which were then incubated at 37 °C for 24 h.

### Sensory evaluation

Sensory analysis was carried out by a panel of 30 individuals, 15 females and 15 males, ages 19 to 60, all non-smokers and regular consumers of dairy products. The sensory attributes of the samples, including flavour, texture, colour and mouthfeel, were rated on a five-point scale, where 0 and 4 meant ’unacceptable‘ and ’really like’, respectively (*19*). Assessors were asked to rinse their mouths with distilled water between samples.

### Statistical analysis

All measurements were performed in triplicate. Data were analysed using the statistical package SPSS v. 20.0 for Windows (SPSS Inc., Chicago, IL, USA). Analysis of variance (ANOVA) was performed for the comparison of physicochemical parameters and probiotic counts among the different experimental groups. The Duncan’s multiple range test was used to determine significant differences. Non-parametric comparisons, including the Kruskal–Wallis test and the Mann-Whitney *U* test, were used for the comparison of sensory values. A significant difference was assessed at p<0.05.

## RESULTS AND DISCUSSION

### Physicochemical properties of ice cream

The results in Table 2 and Table 3 show that the addition of *Bacillus coagulans* did not significantly change the pH and acidity of the different types of ice cream. On the other hand, the addition of fig syrup to ice cream lowered the pH and an increased the acidity of the ice cream, which is consistent with the results of Salama (*20*) and Tammam *et al.* (*21*).

**Table 2:** The pH values of ice cream samples stored at -18 °C for 90 days

Trial	*t*(storage)/day			
1	30	60	90
Control	(6.87±0.03)^a^	(6.65±0.05)^a^	(6.67±0.05)^ab^	(6.54±0.04)^a^
Fig	(6.60±0.06)^b^	(6.35±0.01)^b^	(6.52±0.01)^b^	(6.23±0.07)^b^
Bc	(6.86±0.04)^a^	(6.67±0.04)^a^	(6.80±0.07)^a^	(6.51±0.04)^a^
Fig+Bc	(6.57±0.02)^b^	(6.40±0.01)^b^	(6.60±0.07)^b^	(6.30±0.04)^b^

Mean values (*N*=3) in the same column followed by different lower-case letter in superscript are significantly different (p<0.05). Control=plain dairy ice cream, Fig=ice cream with 25 % fig syrup instead of sugar, Bc=ice cream with *N*(*Bacillus coagulans*)=10^9^ CFU/g as probiotic bacteria, Fig+Bc=ice cream with 25 % fig syrup instead of sugar and *N*(*B. coagulans*)=10^9^ CFU/g

**Table 3:** Titratable acidity in ice cream samples stored at -18 °C for 90 days

Trial	*t*(storage)/day			
1	30	60	90
*γ*(titratable acidity)/(g/L)			
Control	(1.62±0.02)^b^	(1.71±0.01)^b^	(1.66±0.06)^c^	(1.72±0.06)^c^
Fig	(1.75±0.01)^a^	(2.01±0.06)^a^	(1.98±0.03)^a^	(2.11±0.06)^a^
Bc	(1.60±0.04)^b^	(1.65±0.01)^c^	(1.62±0.02)^c^	(1.68±0.01)^c^
Fig+Bc	(1.72±0.05)^a^	(1.98±0.02)^a^	(1.75±0.01)^b^	(2.00±0.03)^b^

Mean values (*N*=3) in the same column followed by different lower-case letters in superscript are significantly different (p<0.05). Control=plain dairy ice cream, Fig=ice cream with 25 % fig syrup instead of sugar, Bc=ice cream with *N*(*Bacillus coagulans*)=10^9^ CFU/g as probiotic bacteria, Fig+Bc=ice cream with 25 % fig syrup instead of sugar and *N*(*B. coagulans*)=10^9^ CFU/g

**Fig. 1:**
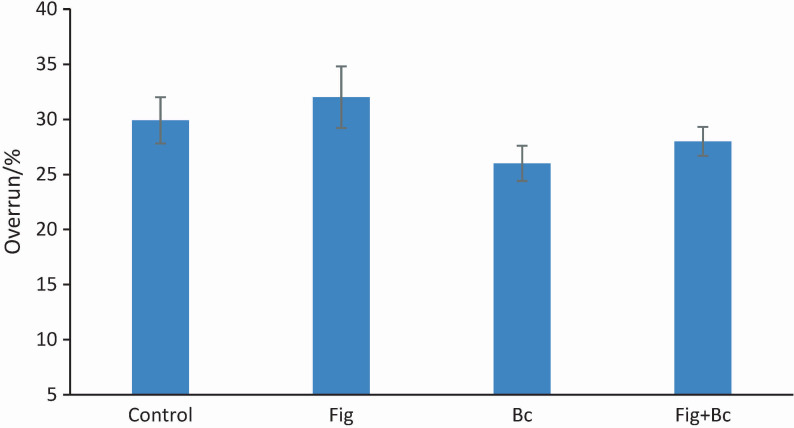
The overrun of ice cream samples immediately after production (mean±SD, *N*=3). Control=plain dairy ice cream, Fig=ice cream with 25 % fig syrup instead of sugar, Bc=ice cream with *N*(*Bacillus coagulans*)=10^9^ CFU/g *Bacillus coagulans* as probiotic bacteria, Fig+Bc=ice cream with 25 % fig syrup instead of sugar and *N*(*B. coagulans*)=10^9^ CFU/g

In agreement with Abghari *et al.* (*22*) and Akalin and Erisir (*23*), the addition of probiotics did not affect the overrun of the probiotic samples. Ghorbani *et al*. (*24*) also reported similar results. Also, the replacement of 25 % sugar with fig syrup did not lead to a significant difference in the overrun of the different types of ice cream. Tammam *et al*. (*21*) reported a significant reduction in overrun by replacing 60 % of the ice cream sugar with date syrup. They explained that the reduction in overrun could be due to inappropriate excessive viscosity, *i.e*. a reduction in viscosity leading to a reduction in the whipping ability of the ice cream mixture. Honey, high-fructose corn syrup (HFCS), corn syrup, sucralose and maltitol have also been reported to reduce overrun (*25*, *26*). The overrun values obtained in the present study were considerably lower than those of commercial ice cream, which could be at least partly due to the ice cream machine used (Fig. 1).

**Fig. 2:**
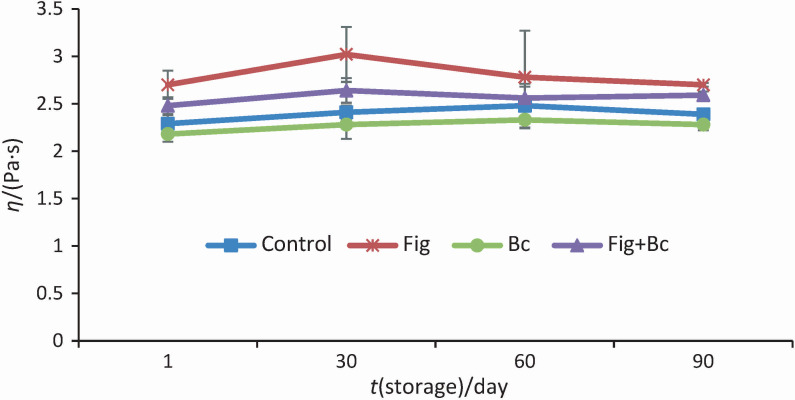
Viscosity of ice cream samples. Control=plain dairy ice cream, Fig=ice cream with 25 % fig syrup instead of sugar, Bc=ice cream with *N*(*Bacillus coagulans*)=10^9^ CFU/g as probiotic bacteria, Fig+Bc=ice cream with 25 % fig syrup instead of sugar and *N*(*B. coagulans*)=10^9^ CFU/g

Apparent viscosity is referred to as the force required to move one layer of fluid over another (*27*). In the present study, the addition of probiotic bacteria to ice cream had no effect on viscosity (Fig. 2). However, replacing sugar with fig syrup resulted in a significant increase in apparent viscosity, which is consistent with previously published results (*21*, *22*, *28*). Fig syrup has a higher water-binding capacity than sugar, possibly due to the pectin content, which has hydrocolloidal properties. Replacing sugar with HFCS, honey and glucose syrup has been shown to have similar effects on the apparent viscosity of ice cream (*25*). In a study by Akalin and Erisir (*23*), the addition of inulin and oligofructose to probiotic ice cream resulted in higher viscosity, which may be due to the interactions between the dietary fibre and the liquid components of the ice cream mixture.

**Fig. 3:**
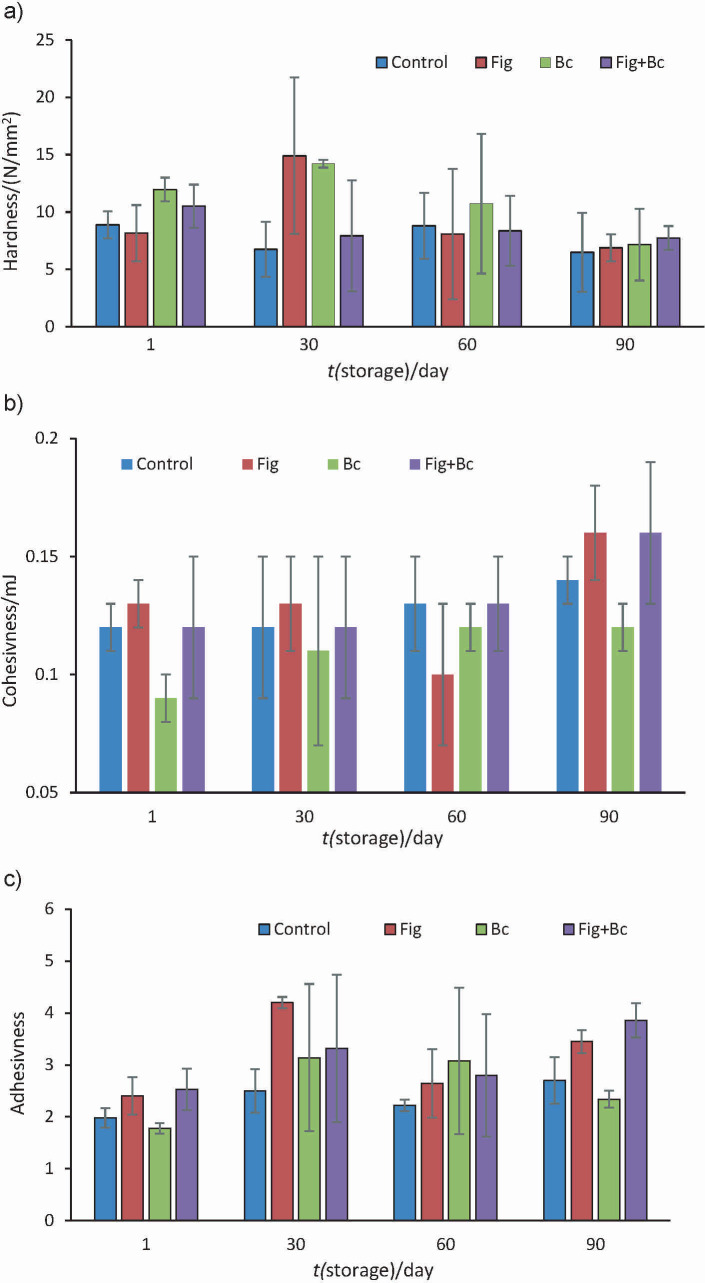
The textural characteristics of the ice cream samples: a) hardness, b) cohesiviness and c) adhesiveness. Control=plain dairy ice cream, Fig=ice cream with 25 % fig syrup instead of sugar, Bc=ice cream with *N*(*Bacillus coagulans*)=10^9^ CFU/g as probiotic bacteria, Fig+Bc=ice cream with 25 % fig syrup instead of sugar and *N*(*B. coagulans*)=10^9^ CFU/g

Replacing 25 % sugar with fig syrup had no effect on the textural properties (except adhesiveness) of ice cream (Fig. 3). In the study by Hashim and Shamsi (*29*), the addition of 50 and 100 % date syrup reduced the hardness values of ice cream samples, while 25 % date syrup had no significant effect on texture. A harder texture was found when sucrose was replaced by maltitol and sucralose.

### Viable counts of probiotic microorganism

The viability of probiotic bacteria can be affected by their initial count, temperature, type of food carrier and storage conditions. Other parameters that may influence probiotic viability include probiotic strain, pH and freezing and thawing conditions (*30*).

**Fig. 4:**
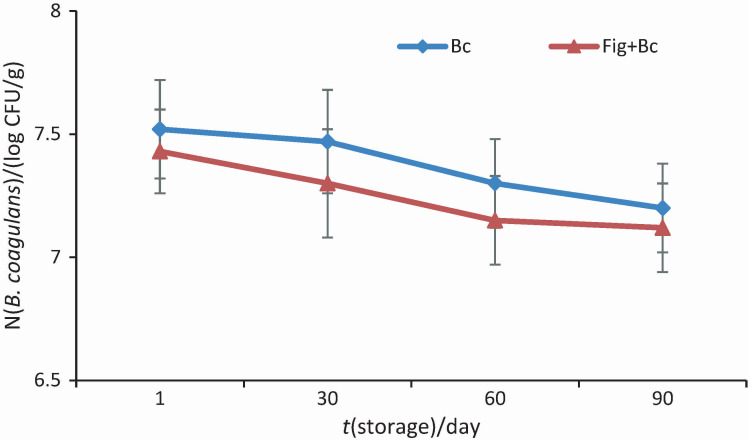
Survival of *Bacillus coagulans* in ice cream samples. Bc=ice cream with *N*(*Bacillus coagulans*)=10^9^ CFU/g as probiotic bacteria, Fig+Bc=ice cream with 25 % fig syrup instead of sugar and *N*(*B. coagulans*)=10^9^ CFU/g

According to the results of bacterial count (Fig. 4), initial freezing of the mixture in a freezer resulted in a significant decrease in probiotic count in all samples, while storage at -18 °C for three months had no significant effect on their survival. Our results are in line with numerous recent studies on the enrichment of dairy products with various microbial strains (*2*, *13*, *24*). Ghorbani *et al*. (*24*) found similar results demonstrating the effect of fortification with iron and *L. casei* on probiotic ice cream properties. Their results showed that the bacterial count in the ice cream samples decreased during storage.

The reduction in the number of probiotic bacteria was only partially related to ice crystal formation and freezing damage; the possible deleterious effects of aeration and mechanical stress during initial freezing should not be ignored (*31*-*33*). Saccharides, especially sucrose, are one of the main ingredients in the production of ice cream. Their cryoprotective properties may enhance the viability of probiotics in frozen products (*27*). The contact of probiotics with the cryoprotective components of the ice cream mixture, such as proteins and sugars, during overnight ripening at 4 °C could play a role in protecting the bacteria during freezing (*22*).

### Sensory properties of ice cream

**Fig. 5:**
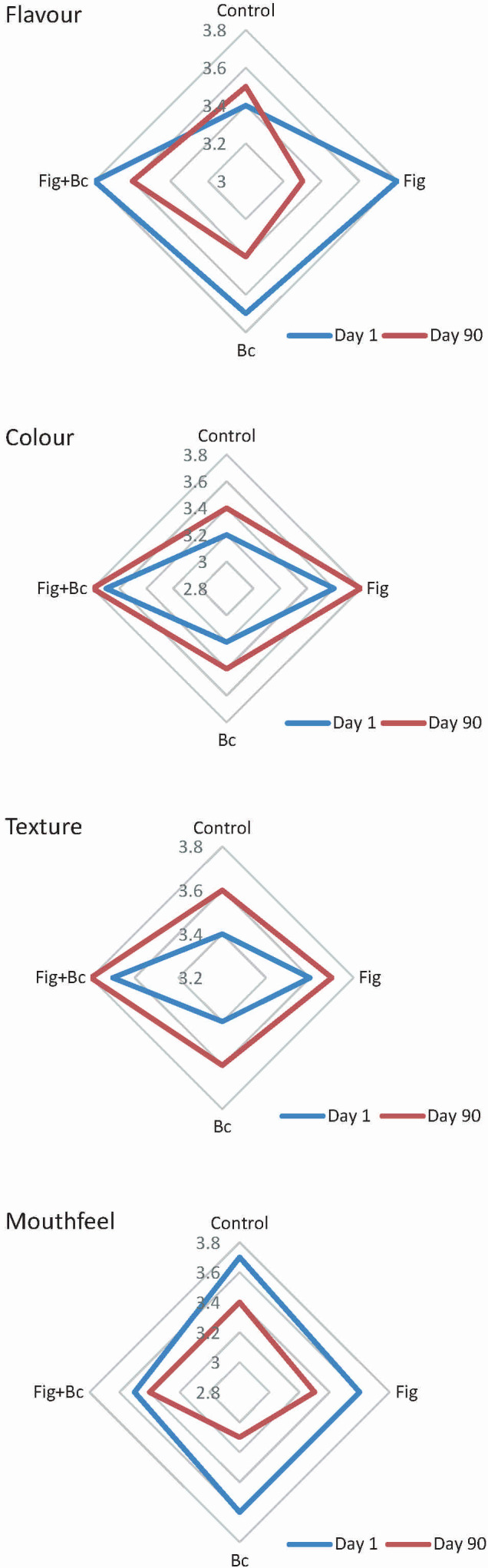
Sensory evaluation of different treatments of ice cream on days 1 and 90 of storage. Control=plain dairy ice cream, Fig=ice cream with 25 % fig syrup instead of sugar, Bc=ice cream with *N*(*Bacillus coagulans*)=10^9^ CFU/g as probiotic bacteria, Fig+Bc=ice cream with 25 % fig syrup instead of sugar and *N*(*B. coagulans*)=10^9^ CFU/g

The results of the sensory analysis are shown in Fig. 5. The addition of *B. coagulans* to ice cream had no negative effect on its organoleptic properties. The addition of fig syrup instead of 25 % sugar gave similar results. All sensory properties (colour, texture, flavour and mouthfeel) were rated above three points in all treatments. Storing the samples in the freezer for 90 days did not lead to any organoleptic complaints. Crumbly, weak, greasy or sandy texture was not observed in any of the treatment groups.

Salama (*20*) and Tammam *et al.* (*21*) used date syrup as a sweetener and flavour ingredient in the production of ice cream. The substitution of date syrup for 40 % sugar resulted in an acceptable product. Salem *et al.* (*34*) showed that enrichment of ice cream with probiotic strains did not affect the acceptability or flavour of the product.

## CONCLUSIONS

The development of ice cream enriched with fig syrup and a probiotic such as *Bacillus coagulans* could be an effective means of increasing the nutritional and functional value of ice cream. In the present study, four formulae for ice cream containing fig syrup and a probiotic strain (*B. coagulans*) were developed. The presence of these functional ingredients did not show any adverse effects on the physicochemical, rheological and sensory properties of the ice cream. The microbial population of *B. coagulans* in the ice creams remained above the threshold of 10^6^ CFU/g even after 90 days of storage at -18 °C. Due to the health benefits of fig syrup and *B. coagulans* probiotic bacteria, the production and consumption of functional ice cream are recommended.
